# Socioeconomic Barriers to Receiving Early Salvage Radiotherapy for Locally Advanced Prostate Adenocarcinoma: A Retrospective Single-Center Study

**DOI:** 10.7759/cureus.68945

**Published:** 2024-09-08

**Authors:** Olivia Heutlinger, Armon Azizi, Garrett Harada, Jeremy P Harris, Michael Daneshvar, Greg Gin, Edward Uchio, Nataliya Mar, Arash Rezazadeh, Steven N Seyedin

**Affiliations:** 1 Radiation Oncology, University of California Irvine School of Medicine, Irvine, USA; 2 Radiation Oncology, University of California Irvine Medical Center, Orange, USA; 3 Urology, University of California Irvine Medical Center, Orange, USA; 4 Hematology and Oncology, University of California Irvine Medical Center, Orange, USA; 5 Hematology and Medical Oncology, University of California Irvine Medical Center, Orange, USA; 6 Radiation Oncology, University of California San Francisco Medical Center, San Francisco, USA

**Keywords:** barriers to radiation, prostate cancer (pca), psa elevation post radical prostatectomy, salvage radiation, socioeconomic factors

## Abstract

Purpose

This study aimed to identify factors associated with delays in initiating early salvage radiation therapy in prostate cancer patients with prostate-specific antigen (PSA) failure after prostatectomy.

Methods

We conducted a single-institution, retrospective study of patients receiving salvage radiation therapy after radical prostatectomy from 2011 to 2022. Patient demographics and clinical data were examined to identify factors that may have influenced the time to start of radiation therapy after surgery. Utilizing a PSA cut off of 0.25 ng/ml or less, we classified patients as receiving either early “PSA low” or late “PSA high” salvage therapy depending on their PSA at the time of initiating treatment.

Results

Of the 81 patients evaluated, the median age was 61.9 years (IQR 57.9 - 66.5), with most presenting with pT3 (65.4%), Grade Group 2 disease (35.8%), and positive margins 55%). Median PSA at salvage radiation therapy commencement was 0.30 ng/mL (0.18 - 0.48). 40 patients completed early salvage and 41 patients completed late salvage in the overall cohort. A significant association was found between patient insurance carrier and pre-radiation PSA levels. Patients with HMO (Health Maintenance Organization) or PPO (Preferred Provider Organization) insurance were more likely to complete late salvage radiation compared to non-managed Medicare patients (HMO OR 4.0, p <0.05 & PPO OR 3.3 p <0.05 vs non-managed Medicare). All uninsured patients in the cohort received late salvage radiation.

Conclusions

Insurance type was significantly associated with the timing of salvage radiation therapy post-prostatectomy, suggesting a relationship with providers requiring prior authorization (HMO and PPO coverage). This study supports proper PSA surveillance, in particular for those with HMO or PPO coverage.

## Introduction

Approximately 288,300 new cases of prostate cancer are diagnosed in the United States each year, with the majority being non-metastatic [1]. Of these patients, a significant number will undergo a robotic radical prostatectomy [2]. Recent trials investigating the timing of radiation after prostatectomy have found that approximately 30% to 50% of prostate cancer patients with high-risk pathologic features, including a positive margin, pathologic T3/4 disease, and Gleason Score of 8 to 10, develop prostate-specific antigen (PSA) relapse, which is defined as sequentially rising PSA or a value higher than 0.2 ng/ml [3-5]. The standard of care for patients who experience PSA failure after radical prostatectomy includes daily salvage radiation therapy over the course of 6.5 to 7 weeks [6].

Studies examining the timing of salvage radiation therapy note that initiation of salvage therapy at a lower PSA impacts outcomes [7,8]. A recent multi-institutional retrospective study consisting of greater than 25,000 patients after prostatectomy noted an elevated all-cause mortality when salvage treatment occurred at PSA greater than 0.25 ng/ml (HR 1.61; 95% CI 1.21 to 2.14). Additionally, prospective trials examining the role of treatment intensification note the need for additional treatment primarily when presenting with a higher PSA at the time of relapse. Re-analysis of Radiation Therapy Oncology Group (RTOG) 9601 appreciated an overall survival benefit with the addition of androgen deprivation therapy (ADT) only when PSA was at least 0.6 ng/ml (HR 0.61, 95% CI, 0.39 - 0.94) [9]. The SPPORT trial found a biochemical failure reduction benefit of elective pelvic radiation therapy only at PSA failure of 0.35 ng/ml and higher [10]. These results emphasize the importance of initiating radiation when PSA is lower, as treatment escalation could be avoided, which is in accordance with recent American Urological Association Guidelines [11].

While multiple studies have examined barriers to receiving definitive radiation treatment for prostate cancer [12-15], none focus on the clinical and demographic factors as barriers to receiving early salvage radiation therapy. The identification of such socioeconomic factors associated with delayed receipt of salvage radiotherapy may identify a patient population that requires increased clinical coordination to ensure they receive standard-of-care therapy. In this study, we aimed to identify these factors.

## Materials and methods

We conducted a single institution retrospective study (IRB# 2021-6819) to identify patients with non-metastatic prostate adenocarcinoma, who completed radical prostatectomy followed by salvage prostate bed radiation therapy for PSA failure from 2011 to 2022. All patients were required to have screening imaging (MRI pelvis, Tm-99 (technetium-99m) bone scan, prostate-specific membrane antigen positron emission tomography (PSMA PET)/CT)) at the time of PSA relapse to rule out metastatic disease. Patients who completed adjuvant radiation therapy were excluded from this study. Initiation of ADT prior to salvage radiation was allowed.

Social and patient demographic factors were extracted from the electronic medical chart, including age, race, primary language, zip code, medical insurance, referring provider, and whether patients were seen by a radiation oncologist before PSA relapse, either at their initial consultation appointment or within 30 days of prostatectomy. Race was categorized as either White, Black, Hispanic, Asian, and Other. Insurance type was categorized into four groups: non-managed Medicare (i.e. Medicare A&B), health maintenance organization (HMO), including Medicaid, preferred provider organization (PPO), and none.

The PSA cut-off of 0.25 ng/ml in this study was selected based on results of a previous multi-center retrospective analysis demonstrating that initiation of salvage radiation therapy at PSA level of greater than 0.25 ng/ml correlated with worse all-cause mortality [7]. In this study, patients were categorized into “PSA low” (aka early salvage) if their PSA was at or below 0.25 ng/ml and “PSA high” (aka late salvage) if their PSA was greater than 0.25 ng/ml at the time of salvage radiation initiation. Additionally, PSA relapse was defined as a PSA of a minimum of 0.2 ng/ml, with a second confirmatory rise [16].

Statistical analyses were performed in R version 4.2.3 (R Foundation for Statistical Computing, Vienna, Austria). To compare continuous variables, a 2-sided Student’s T-test was used for normally distributed variables, and Wilcoxon test was used for non-normally distributed variables. A Fisher’s exact test was used for discrete variables. The Tableone package was used to generate summary tables and statistics. The significance level for this study was defined as p=0.05.

## Results

A total of 81 patients were eligible for study analysis. The median patient age at the time of initiating salvage radiation was 64.6 years old (IQR 61.1 - 69.0) (Table 1).

**Table 1:** Patient Characteristics PPO: Preferred Provider Organization; HMO: Health Maintenance Organization; PSA: prostate-specific antigen; PSMA PET: prostate-specific membrane antigen positron emission tomography; UCI: University of California Irvine; RT: radiation therapy; pT: pathologic primary tumor stage; pN: pathologic nodal stage; R0: margin negative; R1: microscopic margin positive

Total	81
Age at Diagnosis (median [IQR])	61.93 [57.87, 66.48]
Insurance Type (%)	
Medicare	25 (30.9)
PPO	34 (42.0)
HMO	18 (22.2)
None	4 (4.9)
Language (%)	
English	75 (92.6)
Spanish	5 ( 6.2)
Chinese	1 ( 1.2)
pT stage	
2	26 (32.1)
3	53 (65.4)
4	2 (2.5)
pN stage	
0	71 (87.6)
1	10 (12.3)
Margins	
R0	37 (45.7)
R1	44 (54.3)
Gleason Group	
1	4 (4.9)
2	29 (35.8)
3	22 (27.2)
4	9 (11.1)
5	17 (21.0)
PSA before surgery (median [IQR])	9.2 [6.5, 14.7]
Age at Surgery (median [IQR])	62.4 [58.7, 66.9]
Received PSMA PET/CT at relapse	
Yes	58 (71.6)
No	23 (28.4)
Location of PSA Failure	
Biochemical only	38 (66.7)
Bed	9 (15.8)
Lymph node	8 (14.0)
Lymph Node and surgical bed	2 ( 3.5)
PSA at Salvage RT (median [IQR])	0.30 [0.18, 0.48]
Distance to UCI (mi) (median [IQR])	10.3 [6.8, 17.3]
Months From Surgery to Radiation (median [IQR])	13.84 [8.61, 37.22]

The most common pathologic variables at prostatectomy included the pT3 stage (65.4%), Grade Group 2 disease (35.8%), and positive margins (55.0%). The median PSA at the time of salvage radiation therapy initiation was 0.3 ng/ml (IQR 0.18 - 0.48 ng/ml). At the time of PSA failure, 71.6% completed restaging Axumin or PSMA PET/CT imaging. Most recurrences were biochemical only in 72.5% of patients. The median PSA of those with radiographically visible relapse was higher than those with biochemical relapse alone (0.30 vs 0.48 ng/ml; p = 0.31). Fifty (62.5%) patients included were Caucasian and nearly all (92.6%) spoke English. The median distance from the patient listed zip code to the Medical Center was 10.3 miles (IQR 6.8 - 17.3 miles). Patients with early and late salvage radiation were evenly distributed, with 41 patients (50.6%) receiving late salvage radiation. The median time to surgery from salvage radiation in either early salvage or delayed cohorts was similar (13.9 vs 13.2 months).

When examining the association between demographic features and PSA status (as a binary variable stratified by a 0.25 ng/ml cutoff) at the time of salvage radiation, we found a significant association between the patient insurance carrier and pre-radiation PSA level (insurance status vs. PSA level fisher's exact p = 0.017) (Table 2). Patients with PPO or HMO insurance were more likely to be PSA high compared to Medicare patients (PPO: 46.3% vs 37.5%, OR 3.26 [1.08 - 9.83], p<0.05; HMO: 26.8% vs 17.5%, OR 4.04 [1.11 - 14.66], p<0.05). Uninsured patients were more likely to be PSA high than PSA low, although this trend was not statistically significant (9.8% vs 0.0%, N.S.). Differences in age, race, language, or distance to the Medical Center were not appreciated between PSA high and PSA low patients (Table 2).

**Table 2:** Differences in PSA High and Low Cohorts by Socioeconomic Variables PSA Low - patients who started salvage radiation therapy at a PSA of 0.25 ng/ml or less, PSA High - patients who started salvage radiation therapy at a PSA of higher than 0.25 ng/ml, OR: Odds Ratio, IQR: Interquartile Range, PSA: prostate-specific antigen, PPO: Preferred Provider Organization, HMO: Health Maintenance Organization; UCI: University of California Irvine

	PSA Low	PSA High	p	OR	OR CI (95%)
n	40	41			
Age at Diagnosis (median [IQR])	62.7 [59.1, 67.2]	60.4 [56.7, 65.2]	0.12	0.95	0.89 - 1.01
Race (%)					
White	24 (60.0)	26 (65.0)		Reference	
Asian	6 (15.0)	6 (15.0)	0.90	0.92	0.26 - 3.26
Hispanic	4 (10.0)	3 ( 7.5)	0.65	0.69	0.14 - 3.42
Other	5 (12.5)	1 ( 2.5)	0.14	0.19	0.02 - 1.70
Black/African American	1 ( 2.5)	4 (10.0)	0.26	3.69	0.39 - 35.40
Language (%)					
English	38 (95.0)	37 (90.2)		Reference	
Spanish	2 ( 5.0)	3 ( 7.3)	0.65	1.54	0.24 - 9.76
Chinese	0 ( 0.0)	1 ( 2.4)		-	-
Insurance Type (%)			0.02		
Medicare	18 (45.0)	7 (17.1)		Reference	
PPO	15 (37.5)	19 (46.3)	0.04	3.26	1.08 - 9.83
HMO	7 (17.5)	11 (26.8)	0.03	4.04	1.11 - 14.66
None	0 ( 0.0)	4 ( 9.8)		-	-
Age at Surgery (median [IQR])	63.8 [59.8, 67.5]	62.2 [57.5, 65.6]	0.08	0.94	0.88 - 1.01
Age at Radiation (median [IQR])	64.69 [62.04, 69.05]	64.6 [59.0, 68.6]	0.21	0.96	0.91 - 1.02
Distance to UCI (mi) (median [IQR])	10.2 [6.6, 16.1]	10.6 [6.9, 17.5]	0.58	1.01	0.97 - 1.06
Months From Surgery to Radiation (median [IQR])	13.9 [9.3, 26.1]	13.18 [8.35, 46.68]	0.13	1.01	0.996 - 1.024

We next evaluated whether any pathologic features were associated with PSA status at the time of salvage radiation (binary variable). There were no significant associations observed between pathologic tumor stage, lymph node involvement, margin status, grade group, failure location, pre-treatment PSA, PSA doubling time, or Decipher score and PSA status at the time of salvage radiation (Table 3). 

**Table 3:** Differences in PSA High and Low Cohorts by Pathologic Variables LN: Lymph Node, DT: Doubling Time, OR: Odds Ratio, IQR: Interquartile Range, PSA: prostate-specific antigen, RT: radiation therapy; pT: pathologic primary tumor stage; pN: pathologic nodal stage; R0: margin negative; R1: microscopic margin positive

	PSA Low	PSA High	p	OR	OR CI (95%)
n	40	41			
pT stage (%)					
2	10 (25.0)	16 (39.0)		Reference	
3	30 (75.0)	23 (56.1)	0.13	0.48	0.18 - 1.25
4	0 ( 0.0)	2 ( 4.9)		-	-
Nodal Status					
pN0	29 (78.4)	34 (94.4)		Reference	
pN1	8 (21.6)	2 ( 5.6)	0.06	0.21	0.04 - 1.08
Margins					
R0	18 (45.0)	18 (45.0)		Reference	
R1	22 (55.0)	22 (55.0)	1.00	1.00	0.41 - 2.41
Gleason Group (%)					
1	1 ( 2.5)	3 ( 7.3)		Reference	
2	16 (40.0)	13 (31.7)	0.28	0.27	0.03 - 2.92
3	9 (22.5)	13 (31.7)	0.55	0.48	0.04 - 5.40
4	6 (15.0)	3 ( 7.3)	0.19	0.17	0.01 - 2.37
5	8 (20.0)	9 (22.0)	0.43	0.38	0.03 - 4.37
Location of PSA Failure (%)					
Biochemical	15 (68.2)	23 (65.7)		Reference	
Bed	3 (13.6)	6 (17.1)	0.73	1.30	0.28 - 6.03
LN	4 (18.2)	4 (11.4)	0.58	0.65	0.14 - 3.01
LN + Bed	0 ( 0.0)	2 ( 5.7)	-	-	-
PSA before surgery (median [IQR])	9.00 [6.5, 13.8]	9.50 [6.70, 15.5]	0.98	1.00	0.96 - 1.04
Decipher Score (median [IQR])	0.7 [0.4, 0.8]	0.6 [0.3, 0.7]	0.65	0.60	0.06 - 5.6
PSA DT (mo) (median [IQR])	5.7 [3.1, 8.2]	3.6 [2.5, 7.7]	0.90	1.00	0.95- 1.06

Finally, we compared all variables with salvage radiation PSA level as a continuous variable using univariate logistic regression and found that having no insurance was independently associated with having higher salvage radiation PSA levels (beta = 1.5, p < 0.001). No other variables were significantly associated with salvage radiation PSA levels in the continuous variable analysis.

## Discussion

In this manuscript, we performed a retrospective analysis to identify social, demographic, and clinical factors associated with the delivery of early versus late salvage radiation following relapse post-prostatectomy. We demonstrated that patient insurance status, particularly having HMO and PPO insurance, is associated with late salvage radiation compared to non-managed Medicare. Further, we saw that lack of insurance is independently associated with late salvage radiation. Additionally, we found that there may exist an association between earlier evaluation by a radiation oncologist and earlier initiation of salvage radiation. Given that earlier initiation of salvage radiation following relapse is associated with improved outcomes, our results identify key clinical and social factors that should be taken into consideration to identify patients who require a more comprehensive peri-operative evaluation.

We appreciated that patients with HMO and PPO insurance are more likely to receive salvage radiation later compared to patients with non-managed Medicare. We hypothesize that these differences may be related to Medicare patients’ automatic authorization for PSA screening. radiation oncology evaluation and subsequent radiotherapy compared to patients with managed Medicare/Medicaid and other private insurance plans. Consistent with our observations, a recent meta-analysis of 32 articles focused on healthcare disparities noted that patients with Medicaid were less likely to receive radiation therapy for prostate cancer [15]. Notably, to the best of our knowledge, this is the first study to evaluate the impact of insurance on the timing of post-operative salvage radiotherapy in prostate cancer.

Additionally, all uninsured patients received delayed salvage radiation in this study. These results align with large database studies that show uninsured patients with locally advanced prostate cancer were more likely to receive delayed care, present with metastatic disease, and experience increased mortality rates as compared to those with insurance in both univariate and multivariate analysis controlling for other sociodemographic factors [12,17]. There were no significant associations between distance to the Medical Center and the timing of salvage radiation, suggesting that the insurance provider is independently associated with PSA at the time of RT and is not a reflection of other barriers to receiving healthcare. Overall, these results suggest that patients with private insurance or HMO as well as uninsured patients would benefit from closer follow-up and timely care post-prostatectomy. While most patients in our cohort were under age 65 and therefore ineligible for Medicare, providing social assistance to help establish adequate insurance and creating systems to promote timely PSA surveillance after prostatectomy regardless of insurance type should be emphasized to reduce prostate cancer-specific mortality.

The primary limitations of this study are its sample size and retrospective nature. In our cohort, Black patients were more likely to have a higher PSA at the start of salvage radiation therapy (RT). However, again, our sample size was low, and Black patients accounted for 6.2% of the entire study population. As such, it is possible that this trend would be significant in a larger cohort. Larger population studies have found that minority patients are more likely to experience delays in radiation care [12,17]. Additionally, our study did not appreciate a significant correlation between the timing of salvage radiotherapy and income, possibly due to its small sample size. Multiple studies have noted lower income associated with lower PSA screening and less active treatment [15,18]. Another caveat in this study was the choice of utilizing a PSA cutoff of 0.25 ng/ml to define early versus late salvage radiation therapy. While the 0.25 ng/ml level was chosen due to its association with all-cause mortality, some studies recommended a PSA between 0.5 ng/ml and 0.6 ng/ml at the time of salvage RT initiation [9,11].

## Conclusions

This investigation sought to identify factors associated with delays in the initiation of early salvage radiation therapy for patients with PSA failure post radical prostatectomy. We found that insurance type was associated with the timing of salvage radiation therapy, namely non-managed Medicare patients receiving salvage RT at lower PSA values compared to their Medicaid/PPO and uninsured counterparts. Additional associations between race and socioeconomic status did not yield any significant results, likely due to a low study sample size. This study highlights the importance of connecting uninsured patients to receive early salvage radiation therapy.
